# Synergistic effects of biochar and cellulase on carbon emission reduction and humification during co-composting of cattle manure and sugarcane bagasse

**DOI:** 10.3389/fmicb.2026.1803920

**Published:** 2026-04-01

**Authors:** Xinyu Liu, Xiaojian He, Mi Li, Quanbing Pan, Bin Huang, Leye Huang, Jie Wang

**Affiliations:** 1 Pest Integrated Management Key Laboratory of China Tobacco, Tobacco Research Institute of Chinese Academy of Agricultural Sciences, Qingdao, China; 2 Yunnan China Tobacco Industry Co., Ltd., Kunming, China

**Keywords:** biochar, carbon emission reduction, cellulase, humification, microbial community

## Abstract

**Introduction:**

The emission of carbon dioxide (CO₂) and methane (CH₄) during composting not only contributes to the greenhouse effect but also leads to carbon loss, thereby reducing humus production and compromising the quality of the final compost product. This study investigated the efficacy and underlying mechanisms of biochar and cellulase addition, individually and in combination, in mitigating emissions and enhancing compost quality within a cattle manure-sugarcane bagasse co-composting system.

**Methods:**

By monitoring key composting parameters and combining with high-throughput sequencing analysis of bacterial and fungal communities, the changes of microbial structure and function were evaluated, focusing on organic carbon transformation, humification pathway and greenhouse gas mitigation mechanism.

**Results and discussion:**

The results demonstrated that compared with the control, the combined application of biochar and cellulase (particularly the treatment with 5% biochar, denoted as MC5) significantly reduced the cumulative emissions of CO_2_ and CH_4_ by 18.6 and 31.2%, respectively. In addition, the combined treatment promoted the synthesis of humic substances, markedly increased humic acid content by 41.0% (from 28.56 to 40.27 mg/g) and the humification index (HI) by 75.6% (from 2.35 to 4.13). Microbial community analysis revealed that the combined amendment shaped the microbial succession by enriching lignocellulose-degrading fungi (such as *Mycothermus*) and humification-promoting bacteria (e.g., *Chryseolinea*), while suppressing taxa associated with greenhouse gas emissions (e.g., *Clostridium*), thereby directing a greater carbon flux toward humification pathways. This study confirms that the synergy between biochar and cellulase effectively mitigates greenhouse gas emissions and improves the quality of compost, providing a theoretical basis for developing low-carbon, high-value composting technologies.

## 1 Introduction

The intensification of global livestock farming has led to the massive accumulation of animal manure, posing a severe environmental challenge. As a major waste stream, improper disposal of cattle manure not only occupies land resources but also contributes to greenhouse gas emissions, water eutrophication, and the spread of pathogens (Bruinenberg et al., 2023; Shaibur et al., 2025). Similarly, sugarcane, as a significant economic crop, generates large quantities of bagasse as a by-product during processing, creating substantial disposal pressures. Aerobic composting represents an economically viable and effective biological treatment technology capable of converting such organic solid wastes into stable and harmless mature compost, thereby facilitating waste resource recovery (Yang et al., 2024). However, conventional composting processes are often plagued by two critical issues: substantial carbon loss (primarily in the form of CO₂ and CH₄) and a low degree of humification. These shortcomings not only exacerbate the greenhouse effect but also compromise the quality and agronomic value of the final compost product (Liu N. et al., 2024; Liu S. et al., 2024; Liu Y. et al., 2024; Peng et al., 2022).

Carbon loss during composting primarily stems from the rapid mineralization of organic matter, during which microorganisms generate substantial amounts of gaseous products (Geng et al., 2024). A pivotal challenge in composting research is how to effectively retain carbon and direct it toward the pathway of stable humus synthesis. In recent years, biochar has been widely employed in composting remediation studies due to its porous structure, high specific surface area, and abundant surface functional groups (Dang et al., 2024; Gu et al., 2024; Zhou et al., 2022). Research indicates that biochar possesses a strong adsorption capacity, with its adsorption capacity for furfural compounds reaching 258.94 ± 3.2 mg/g (Bhatia et al., 2023). Dang et al. (2024) found that adding biochar improves the porosity of the compost pile, promoting an aerobic environment, while its surface properties provide habitats for microorganisms, thereby facilitating the transformation and retention of organic matter and reducing greenhouse gas emissions. Meanwhile, cellulase can directly degrade the cellulose components in compost into utilizable sugars, substituting for part of the microbial decomposition function. These sugars serve not only as an energy source for microbial activity but also as crucial carbon skeletons for synthesizing humus precursors, such as phenols and quinones (Chang et al., 2023). Sun et al. (2021) found the exogenous addition of cellulase accelerated the decomposition of waste mushroom substrate, supplied more precursors for humification, and promoted compost maturation and humification.

The combined application of biochar and enzymes has been successfully implemented in environmental remediation, demonstrating notable synergistic effects (Zhang B. et al., 2025). Enzyme immobilization on biochar enhances stability against pH and thermal variations, improves storage longevity, and consequently increases the remediation efficiency for pollutants (Yang et al., 2025). However, research on this combination in the field of composting remains limited. Existing studies are confined to biochar with laccase, which was shown to accelerate the degradation of artificial sweeteners during sewage sludge composting, or with “garbage enzyme” to enhance humification. Research specifically on the impact of biochar combined with cellulase on composting is absent. Cellulase efficiently breaks down cellulose macromolecules, generating abundant soluble carbon sources. Biochar, with its large specific surface area, can adsorb and protect these intermediate products from rapid mineralization while providing a favorable micro-environment for microbial communities and enzymatic reactions. This synergy is hypothesized to direct carbon flow more effectively toward the humification pathway, offering the potential to significantly enhance compost humification while reducing carbon gas emissions (Jindo et al., 2025). Nevertheless, the synergistic efficacy and underlying mechanisms of biochar and cellulase within composting systems are still not sufficiently understood.

Although biochar and cellulase have been individually applied in composting, their combined effect on regulating mineralization and humification pathways remains unexplored. Specifically, it is unclear whether the synergistic action of cellulase and biochar can effectively divert organic carbon from greenhouse gas emissions toward humic substance synthesis. Addressing this mechanistic gap is essential for developing next-generation composting strategies that simultaneously achieve carbon emission reduction and compost quality enhancement.

Therefore, in this work, we aim to systematically investigate the individual and combined effects of biochar and cellulase on the co-composting process of cattle manure and sugarcane bagasse. The research will focus on evaluating their dynamic impact on greenhouse gas (CO₂ and CH₄) emissions. Furthermore, by analyzing changes in humic substance components (humic acid and fulvic acid) and the microbial community structure, we seek to elucidate the underlying mechanisms by which these amendments promote humification. This study is expected to provide a crucial theoretical foundation and practical strategies for developing efficient, low-carbon, and high-quality composting technologies for agricultural waste.

## 2 Materials and methods

### 2.1 Composting experiment setup

In this study, six treatments were established, including: CK (control), biochar alone (5%, w/w), cellulase alone (10 mL/pile), and combinations of biochar (1, 5, and 10%, w/w) with cellulase. Cattle manure and bagasse were selected as the primary composting materials. The physicochemical properties of the raw materials used in this study are presented in Table 1. These materials were thoroughly mixed at a dry weight ratio of 7:3, and the C/N ratio (approximately 27) and moisture content (approximately 70%) were adjusted to support optimal microbial activity during composting. The bagasse was obtained from a sugarcane processing plant as a by-product after juice extraction. Cattle manure was collected from a local cattle farm, and biochar derived from Sesbania was purchased from Straw Char Technology Co., Ltd. The physicochemical properties of the raw materials are provided in Supplementary Table S1. The commercial cellulase preparation used was Cellic^®^ CTec 3 HS (Novozymes).

**Table 1:** Physicochemical properties of the composting raw materials.

Material	TC (g/kg)	TN (g/kg)	Moisture content (%)	*C*/*N*
Bagasse	46.20 ± 0.21	0.3223 ± 0.05	27 ± 1.23	143.34 ± 8.32
Cattle manure	36.26 ± 3.91	8.33 ± 0.46	27.67 ± 2.52	25.25 ± 2.60
Biochar	60.62 ± 4.95	0.629 ± 0.12	5.4 ± 0.15	60.62 ± 1.50

The experiment was conducted at the Jimo Experimental Station of the Tobacco Research Institute, Chinese Academy of Sciences. Each composting pile was established in a foam box with dimensions of 60 cm × 40 cm × 50 cm, resulting in a total working volume of 10 L per pile. All treatments were initiated on the same day and regularly turned. Due to continuous rainfall at the beginning of the experiment, the ambient temperature was low, resulting in a slow initiation of the composting process. Sampling was performed on days 0, 1, 5, 10, 20, 35, and 38 during the turning process. Approximately 500 g of sample was collected from multiple layers within each pile using a multi-point sampling method. The fresh samples were thoroughly homogenized and stored at −20 °C for subsequent analysis.

### 2.2 Analysis of composting-related parameters

Key maturity indices of the compost, including temperature, pH, electrical conductivity (EC), ammonium nitrogen (NH₄^+^-N), nitrate nitrogen (NO₃^−^-N), and germination index (GI), were determined following the method described by Zhao et al. (2023). Gas samples for CO₂ and CH₄ analysis were collected using the static chamber method on days 0, 1, 5, 10, 20, 35, and 38 of the composting process. Sampling was performed at a fixed time each day to minimize diurnal variation. The concentrations of CO₂ and CH₄ were immediately determined using a gas chromatograph (Agilent 6,890 N Network GC system, Agilent Technologies, United States) equipped with a flame ionization detector (FID) and a thermal conductivity detector (TCD). The injection port, oven, and detector temperatures were set at 120 °C, 60 °C, and 250 °C, respectively. High-purity nitrogen was used as the carrier gas at a flow rate of 30 mL/min. The emission fluxes were calculated based on the rate of change in gas concentration within the chamber over time, following the method described by Wu et al. (2024a,b). Organic matter (OM) content was analyzed by the potassium dichromate volumetric method. Dissolved organic carbon (DOC) was measured with a TOC (total organic carbon) analyzer (Shimadzu TOC-L CPH). Humic substance (HS) was extracted from compost samples using alkaline sodium pyrophosphate. Humic acid (HA) and fulvic acid (FA) were separated by acid precipitation, and the organic carbon contents of HS, HA, and FA were quantified with the TOC analyzer to represent their respective concentrations. All experiments were performed in triplicate.

### 2.3 High-throughput sequencing analysis

Total genomic DNA of the microbial community was extracted from the compost samples using the E. Z. N. A.^®^ Soil DNA Kit (Omega Bio-tek, Norcross, GA, United States). The purity and concentration of the DNA were assessed by 1.0% agarose gel electrophoresis and a NanoDrop2000 spectrophotometer (Thermo Scientific, United States). The V3–V4 region of the bacterial 16S rRNA gene was amplified with the universal primers 338F (5′-ACTCCTACGGGAGGCAGCAG-3′) and 806R (5′-GGACTACHVGGGTWTCTAAT-3′). The fungal ITS region was amplified using the primers ITS5 (5′-GGAAGTAAAAGTCGTAACAAGG-3′) and ITS2 (5′-GCTGCGTTCTTCATCGATGC-3′). Sequencing was subsequently performed on an Illumina HiSeq platform at Majorbio Bio-Pharm Technology Co., Ltd. (Shanghai, China). Raw reads were processed and subjected to quality control in QIIME to obtain clean reads. Sequences with ≥97% similarity were clustered into the same operational taxonomic units (OTUs). All raw sequence datasets generated in this study have been deposited in NCBI Sequence Read Archive under accession number PRJNA1418375.

### 2.4 Statistical analysis

The data were analyzed using the Data Processing System v.6.55 software (Hangzhou RuiFeng Information Technology Co., Ltd., Hangzhou, China). Mean differences were evaluated using Tukey’s honest significant difference (HSD) test at a significance level of *p* ≤ 0.05. Redundancy analysis (RDA) was performed in R package vegan (version 2.15.3). Heatmaps were generated using TBtools (version 1.106).

## 3 Results and discussion

### 3.1 Changes in basic physicochemical parameters during composting

**Figure 1:**
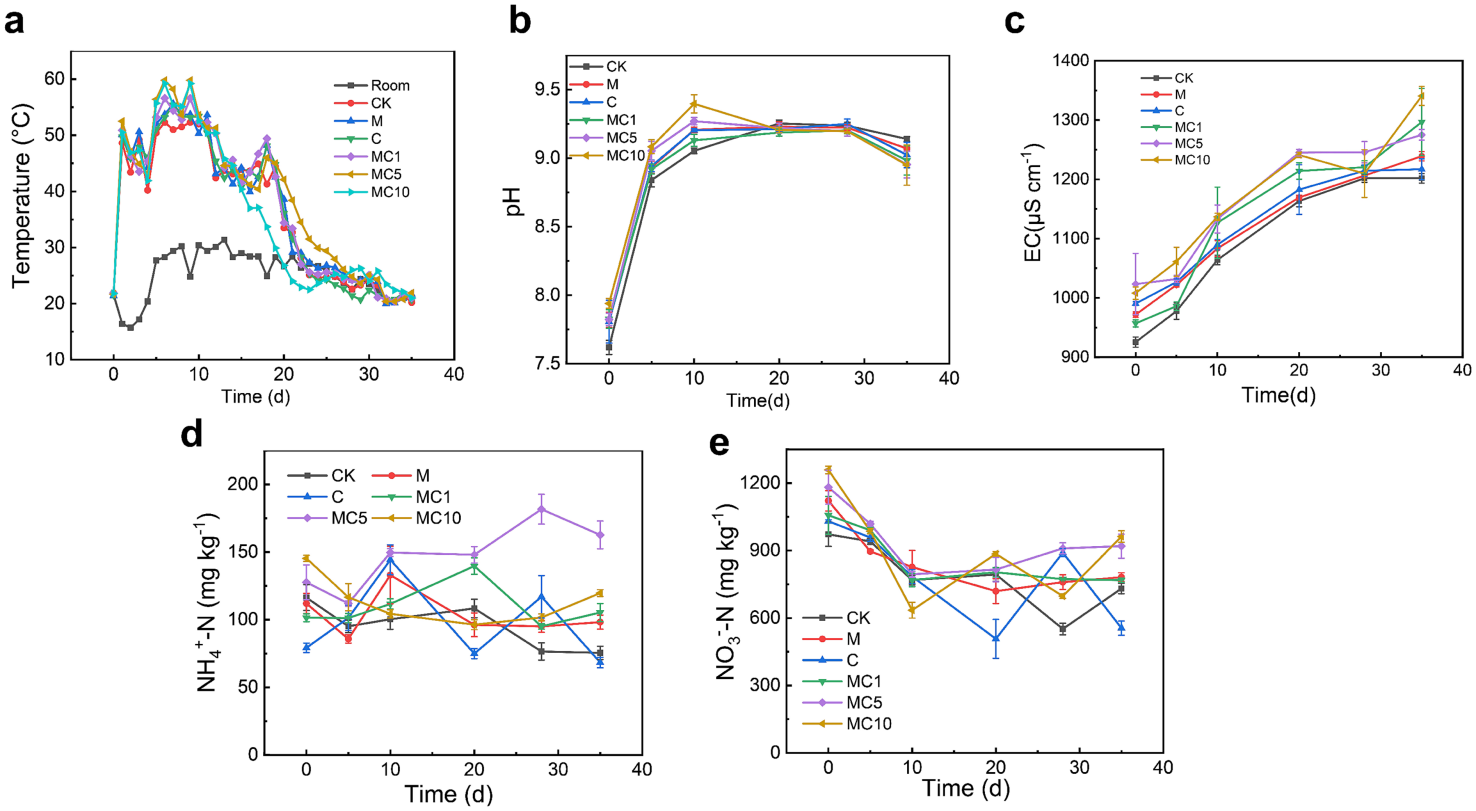
Variations in **(a)** temperature; **(b)** pH; **(c)** electrical conductivity; **(d)** NH_4_^+^; and **(e)** NO_3_^−^ during the composting process.

In this study, both biochar and cellulase treatments enhanced the heating rate and maximum temperature during composting, with the combined biochar-enzyme treatments exhibiting the most pronounced effects. As shown in Figure 1a, after initial fluctuations in the first 3 days due to low ambient temperature, the temperature began to rise rapidly from the fourth day onward. The control (CK) reached 50.4 °C on day 5, while the biochar-only and cellulase-only treatments reached 51.1 °C and 52.1 °C, respectively. In contrast, the combined treatments achieved higher temperatures of 53.2 °C (MC1), 56.4 °C (MC5), and 55.7 °C (MC10). The maximum temperature in the CK was 52.3 °C (on day 6), compared to 55.6 °C (day 7) for the cellulase-only treatment and 55.4 °C (day 7) for the biochar-only treatment. All combined treatments peaked on day 7, reaching 56.6 °C (MC1), 59.8 °C (MC5), and 59.2 °C (MC10). These results indicate that the combined application of biochar and cellulase positively influenced the composting process by accelerating the heating rate and raising the peak temperature. The higher temperatures promoted the degradation of organic matter and enhanced compost maturity, while also contributing to the elimination of pathogenic microorganisms, thereby meeting the sanitization requirements of the final compost product (Tian et al., 2024).

As illustrated in Figure 1b, the pH values in all compost groups initially increased and then decreased, remaining overall alkaline. This pattern is mainly attributed to the rapid microbial decomposition of readily degradable organic materials such as proteins, amino acids, and urea, which release alkaline compounds. In the initial phase, the pH increased more rapidly in the combined treatments than in the single amendment and CK groups, reaching 9.13 (MC1), 9.27 (MC5), and 9.39 (MC10) by day 10. This trend was consistent with the temperature profiles and may be related to the enhanced microbial activity and accelerated organic matter decomposition under the combined treatments. The subsequent decline in pH was associated with acid production by microbial metabolism during the maturation phase, as well as nitrogen transformations such as nitrification and denitrification (Wang J. et al., 2025). Moreover, the pH decrease was more pronounced in the combined treatments than in the single amendment and CK groups, possibly due to enhanced nitrification converting NH₄^+^-N into NO₃^−^-N. Huang et al. (2025) reported that biochar can activate denitrifying bacteria and promote denitrification. Additionally, alkaline conditions have been shown to favor the enrichment of denitrifying bacteria (Xu et al., 2021).

The changes in electrical conductivity (EC) over time are shown in Figure 1c. EC continuously increased throughout the composting process, reaching values between 1,201 and 1,304 μS/cm by day 35. All values remained below 4.3 mS/cm, meeting the maturity criteria for compost (Liu N. et al., 2024; Liu S. et al., 2024; Liu Y. et al., 2024). The sustained rise in EC can be attributed to the accumulation of soluble ions released during microbial decomposition of organic matter. Since the composting systems were largely closed (except for gas exchange), ionic losses were minimal, leading to continuous EC increase (Zhang et al., 2024). Furthermore, due to the high electrical conductivity of biochar, the EC values in biochar-amended treatments were significantly higher than those without biochar (You et al., 2024). The combined treatments enhanced the decomposition efficiency of organic matter, resulting in noticeably higher EC values compared to single amendment and CK groups. The final EC values were 1,201 μS/cm for CK and 1,304 μS/cm for MC10.

NH₄^+^-N and NO₃^−^-N are key nitrogen forms that support microbial metabolism and growth. As shown in Figures 1d,e, except for the MC10 treatment, NH₄^+^-N concentrations remained within a dynamically stable range of 75–150 mg/kg. In the MC10 treatment, NH₄^+^-N content showed a continuous increasing trend, rising from 127 mg kg to 182 mg/kg during composting. At the end of composting, NH₄^+^-N levels in all combined treatments were significantly higher than those in single amendment and CK groups. This also explains why the EC and pH values were higher in the combined treatments. In the early composting stage, microbial decomposition of nitrogenous organic matter released NH₃, part of which dissolved in water, increasing NH₄^+^ levels. Subsequent microbial uptake and nitrification converted part of the NH₄^+^ into NO₃^−^, leading to a decrease in NH₄^+^. The interplay of these processes resulted in a generally stable dynamic range of NH₄^+^-N concentrations. In contrast, NO₃^−^-N content decreased rapidly in the initial stage and stabilized within a dynamic range in the later phase. The early stage was characterized by vigorous microbial growth and intense organic matter decomposition. The abundant carbon sources and elevated temperatures significantly promoted denitrification while inhibiting nitrification, leading to substantial consumption of nitrate nitrogen (González et al., 2024). In the later stage, denitrification weakened, and nitrification intensified. Meanwhile, a balance was reached between microbial assimilation and nitrogen release, ultimately stabilizing the nitrate nitrogen content.

### 3.2 Organic matter degradation and CO₂/CH₄ emissions during composting

**Figure 2:**
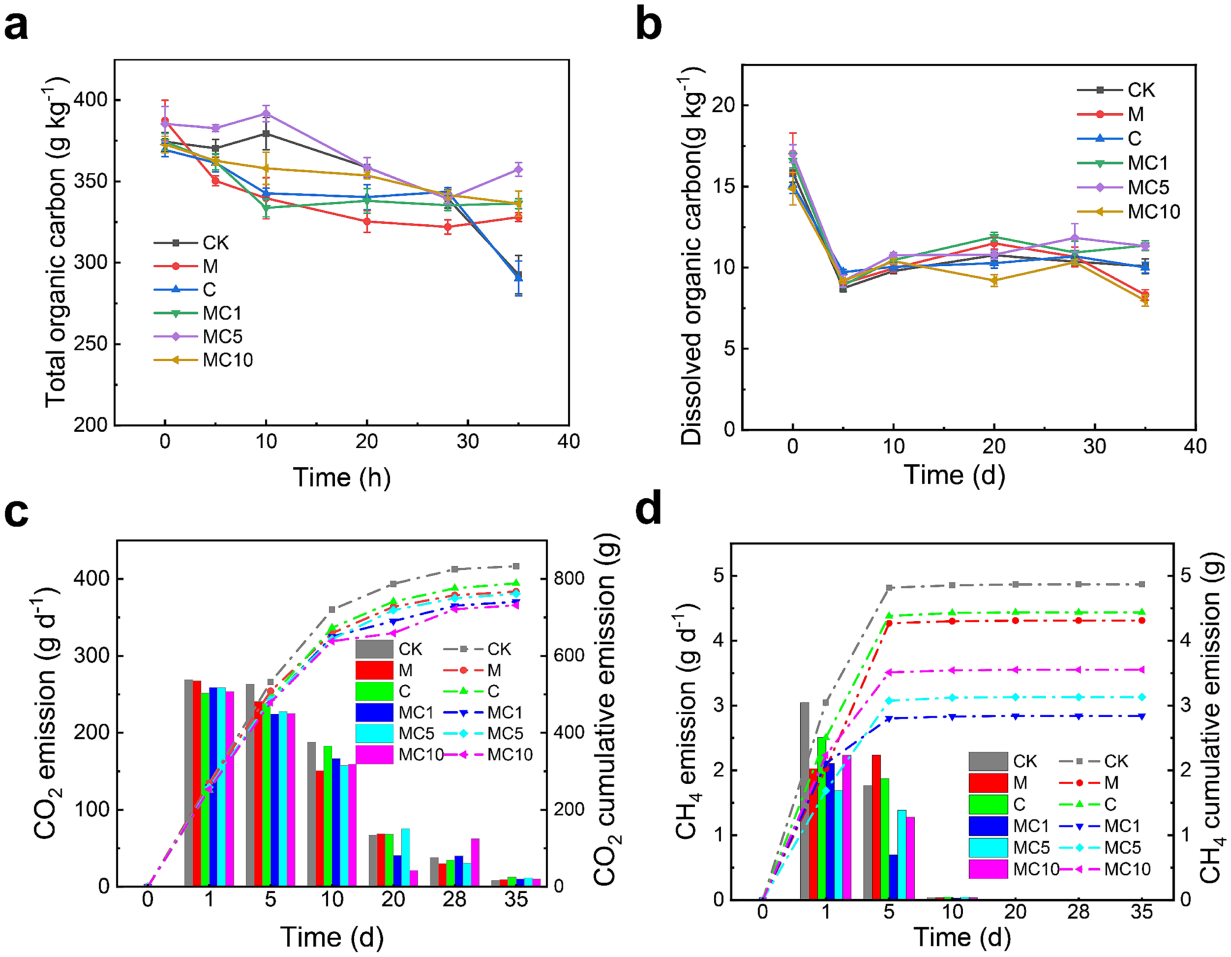
Changes in TOC **(a)**; DOC **(b)**; CO_2_ emission **(c)**; and CH_4_ emission **(d)** during composting.

During composting, microorganisms decompose macromolecular organic matter through respiration, releasing a substantial amount of CO₂ and thereby obtaining energy to sustain their metabolic activities and further promote continuous organic matter degradation (Liu N. et al., 2024; Liu S. et al., 2024; Liu Y. et al., 2024). As shown in Figure 2a, the TOC content of all treatments gradually decreased throughout the composting process, indicating continuous organic matter degradation. This is likely because composting microbes rapidly decomposed readily degradable organic materials in the initial stage, releasing carbon dioxide (Ahn et al., 2024). At the end of composting, the TOC content in the combined enzyme-biochar treatments (MC1, MC5, and MC10) was significantly higher than in the enzyme-only, biochar-only, and CK treatments. The addition of cellulase may partially substitute for microbial functions, reducing some CO₂ emissions. Simultaneously, biochar can adsorb soluble organic matter, reducing reaction intensity and thus lowering CO₂ emissions. This aligns with the findings of Jiang et al., who observed the least carbon loss in compost with combined biochar and garbage enzyme amendment (Jiang et al., 2022). The percentage TOC losses by the end of composting were 21.81% (CK), 15.32% (M), 21.39% (C5), 10.15% (MC1), 7.27% (MC5), and 9.75% (MC10), respectively.

The DOC largely consists of organic matter that can be rapidly utilized by microorganisms. As shown in Figure 2b, DOC content decreased rapidly in the early composting stage and stabilized later. The initial sharp decline is attributed to the rapid consumption of DOC as an easily degradable carbon source by microbes. In the later stage, the proportion of recalcitrant organic matter increased, and the degradation rate slowed, leading to stable DOC levels. Comparing the treatments, the combined enzyme-biochar groups (MC1 and MC5) exhibited higher stabilized DOC values in the later phase, reaching 11.33 and 11.36 g/kg by day 35, respectively, compared to only 10.08 g/kg in the CK group. This further supports that the synergy between enzyme and biochar can degrade recalcitrant organic matter more efficiently, releasing more DOC and providing sufficient energy and substrates for microbial activity and humification (Chang et al., 2023).

As shown in Figure 2c, CO₂ emissions were high during the initial composting phase (0–5 days) and gradually declined thereafter, consistent with the pattern of microbial activity. Early on, strong microbial activity and intense organic matter decomposition produced large amounts of CO₂, while decreased degradation rates in the later stage led to reduced CO₂ emissions. In terms of cumulative emissions, the combined enzyme-biochar treatments showed lower peak CO₂ emissions and lower cumulative emissions, whereas the enzyme-only (M) and biochar-only (C) treatments exhibited relatively higher emissions. Biochar likely adsorbs some easily degradable carbon sources within its pores, reducing their bioavailability. This limits rapid microbial access to sufficient substrates for intensive respiration, thereby reducing immediate CO₂ release. Additionally, cellulase participates in cellulose decomposition, reducing the microbial effort required to break down cellulose, which also contributes to lowering CO₂ emissions.

CH₄ emissions were primarily concentrated in the early composting stage (0–10 days, Figure 2d) and approached nearly zero in the later phase. In the initial stage, the raw materials contained abundant readily degradable organic matter, leading to highly active microbial metabolism that consumed large amounts of oxygen and created localized anaerobic conditions. Methanogens utilized carbon dioxide, acetic acid, and other substances under these conditions, converting them into CH₄. In the later stage, easily degradable organic matter was largely depleted, microbial activity slowed, aerobic conditions dominated, and methanogens became inactive, leading to nearly zero CH₄ emissions. Comparing the treatments, the combined enzyme-biochar groups (MC1, MC5, and MC10) showed relatively lower peak and cumulative CH₄ emissions, while the enzyme-only, biochar-only, and CK groups had higher emissions. These results are consistent with the study by Zhou et al. (2022) on co-composting with biochar and manganese ore (Du et al., 2024).

### 3.3 Humification and maturity changes during composting

**Figure 3:**
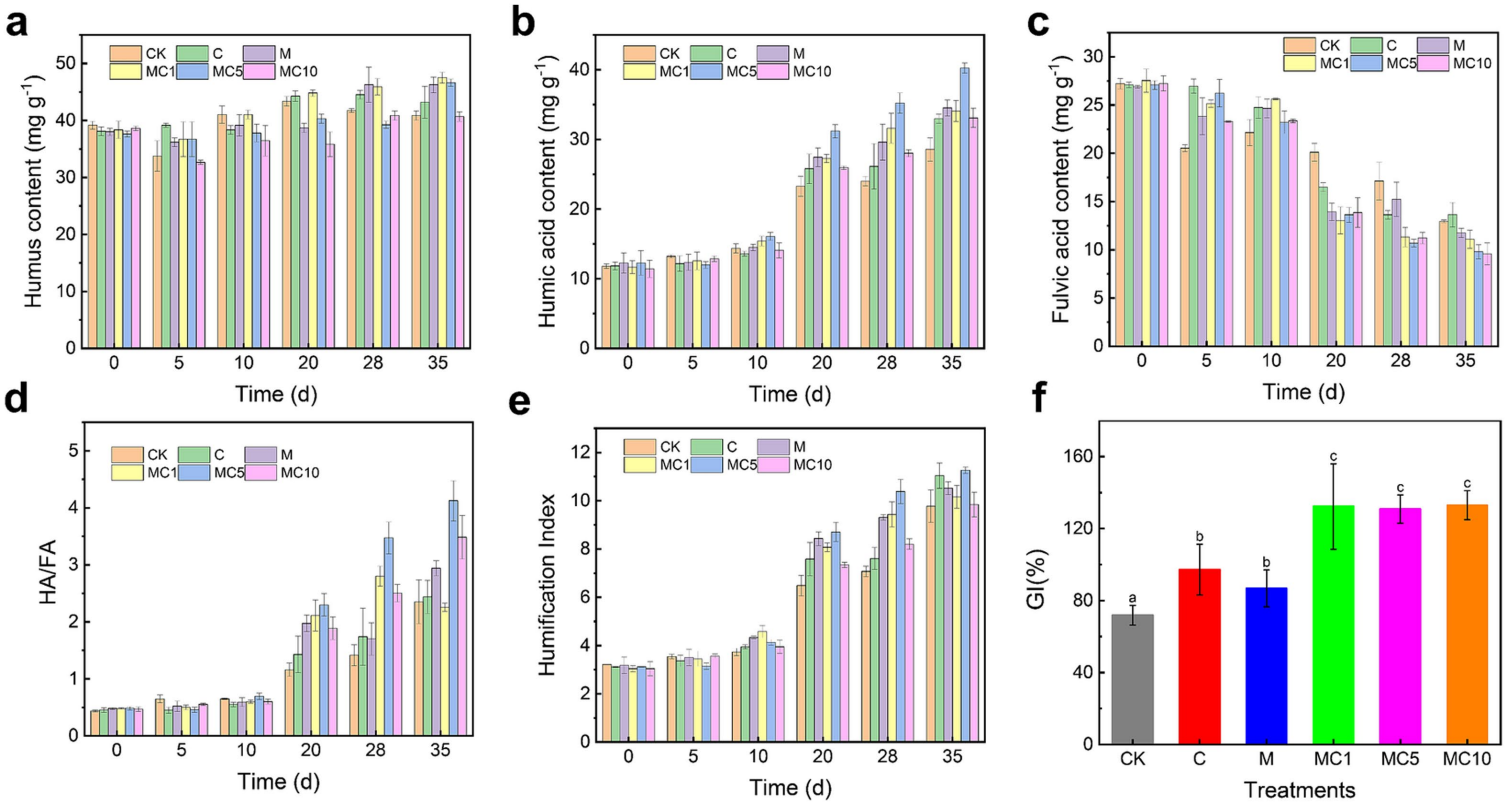
Temporal variations in humus-related parameters during composting: **(a)** HC, **(b)** HA, **(c)** FA, **(d)** HA/FA ratio, **(e)** HI, and **(f)** GI.

Promoting humification is a key process in aerobic composting. Merely understanding the changes in organic matter content does not provide an objective or comprehensive view of material transformation or the progression of composting. Therefore, we investigated the changes in humic substances during composting (Liu et al., 2023). As shown in Figure 3a, the humus content exhibited an increasing trend throughout the process. Significant differences were observed among treatments, with the combined enzyme-biochar groups, especially MC1 and MC5, showing significantly higher humic acid content in the later stages compared to the enzyme-only, biochar-only, and CK treatments. On day 35, the humus contents in MC1 and MC5 were 47.52 and 46.62 mg/g, respectively, while those in the enzyme-only, biochar-only, and CK treatments were only 46.62, 43.20, and 40.86 mg/g. These results indicate that the combined addition of enzyme and biochar promotes humification during composting. Biochar addition can effectively stimulate humus formation by selectively regulating the fungal community and metabolic characteristics during composting (Guo et al., 2024). Cellulase enhances compost maturity and quality by improving the decomposition of waste mushroom substrate (Sun et al., 2021).

Humic substances consist of soluble fulvic acid (FA) and insoluble humic acid (HA). HA plays an important role in soil improvement and creating a favorable crop growth environment. As shown in Figure 3b, HA content gradually increased during composting, while FA content decreased (Figure 3c), consistent with typical aerobic composting trends (Cao et al., 2023). The MC5 treatment showed a more pronounced decrease in FA content in the later stage, along with higher HA content compared to the enzyme-only, biochar-only, and CK treatments. On day 35, the HA content in MC5 was 40.27 mg/g, significantly higher than that in CK (28.56 mg/g), biochar-only (32.99 mg/ g), and enzyme-only (34.51 mg/g) treatments. This suggests that the combined application of enzyme and biochar more effectively promotes the conversion of FA to HA. Furthermore, the amount of biochar added played a critical role in the enzyme-biochar system, as either too much or too little biochar was unfavorable for humus conversion.

Composting is typically a process involving the decomposition and stabilization of organic materials to form polymerized humic substances. The humification degree (HD), humification index (HI), and germination index (GI) are important indicators for assessing the extent of humification and the maturity of the final compost product. As shown in Figure 3d, HD increased during composting, with final values as follows: CK (2.35), C (2.43), M (2.94), MC1 (2.25), MC5 (4.13), and MC10 (3.48). These results indicate that the combined enzyme-biochar addition more effectively promoted the polymerization of humic substances. Moreover, an appropriate biochar dosage was essential to enhance polymerization in the enzyme-biochar system.

Figure 3e depicts a gradually increasing trend in HI across all six treatments. After composting, the MC5 treatment showed the highest HI value among all groups. The HI trend was similar to that of HD (Figure 3e), with each treatment showing a continuous increase throughout the process. In the final compost product, the GI values of MC1, MC5, and MC10 were 132, 131, and 133%, respectively, significantly higher than those of the biochar-only (97%), enzyme-only (87%), and CK (72%) treatments (*p* < 0.05) (Figure 3f). These results demonstrate that the combined use of cellulase and biochar significantly promotes humic substance polymerization and enhances compost maturity.

### 3.4 Succession of microbial communities during composting

**Figure 4:**
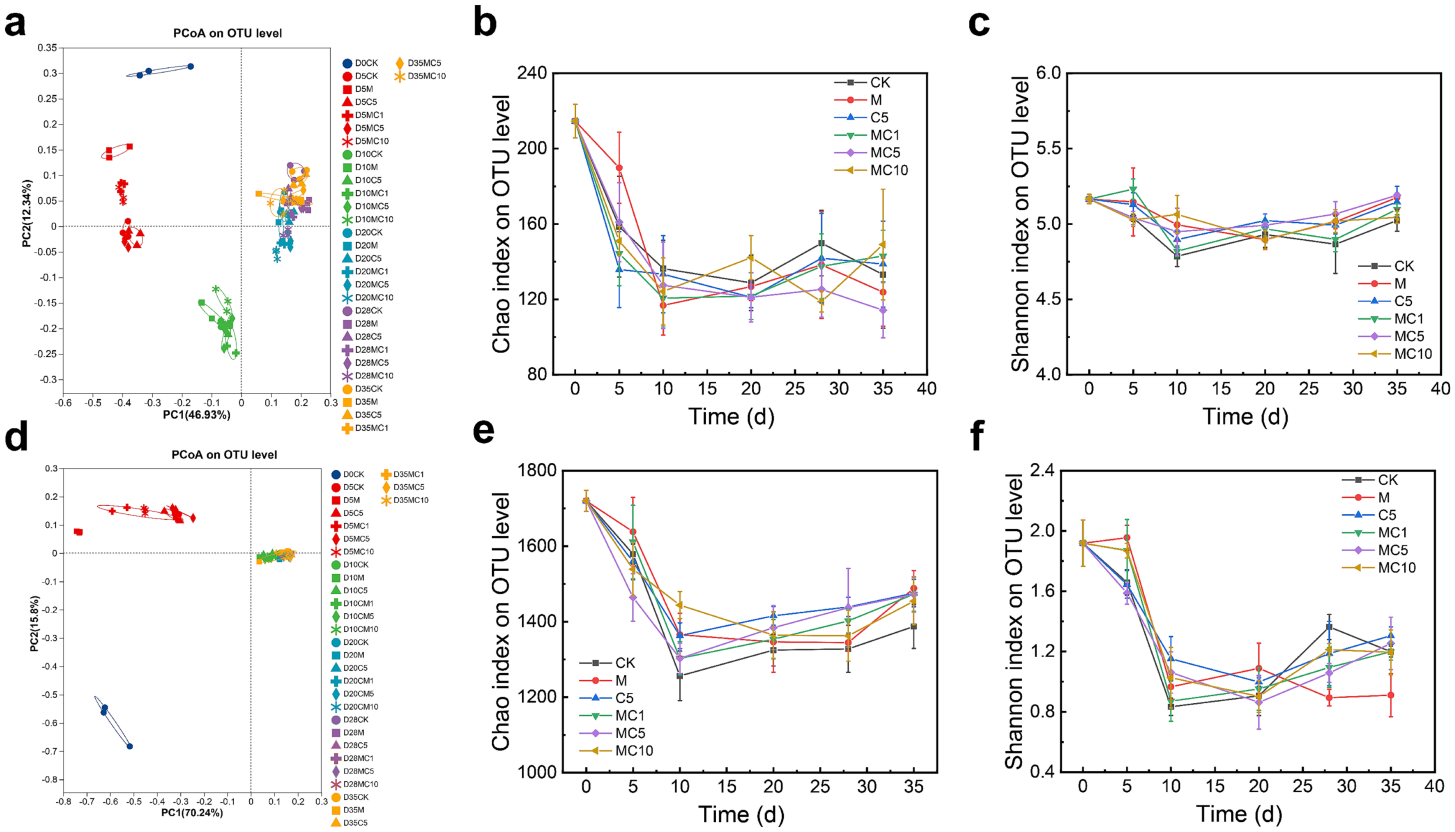
Succession of bacterial and fungal communities during composting: PCoA **(a)**, Chao1 **(b)**, and Shannon **(c)** indices of bacterial communities; PCoA **(d)**, Chao1 **(e)**, and Shannon **(f)** indices of fungal communities.

Composting is fundamentally a natural biochemical process driven and governed by microorganisms, which play a decisive role throughout (Wang et al., 2023). Understanding the succession and variation of microbial communities during composting is essential for elucidating the synergistic mechanisms by which cellulase and biochar reduce carbon emissions and enhance humification. The differences and similarities in microbial community structure are shown in Figures 4a,d. Principal coordinate analysis (PCoA) revealed clear temporal shifts in both bacterial and fungal communities during the initial 10 days. In the later stages, fungal communities stabilized, while bacterial communities continued to exhibit considerable variation. A similar pattern was observed across treatments: both fungi and bacteria showed clear differences early on, but by the later phase, fungal communities became more uniform, whereas bacterial communities remained distinct. We further assessed microbial richness and diversity using the Chao1 (Figures 4b,e) and Shannon indices (Figures 4c,f). Both bacterial and fungal richness and diversity initially decreased, then gradually increased and eventually stabilized. This indicates that although overall microbial diversity declined as composting progressed, the abundance of specific microbial groups increased. The initial high temperatures likely eliminated many non-thermotolerant microorganisms, allowing thermotolerant species to flourish, thereby reducing diversity. As temperatures decreased after the thermophilic phase, non-thermophilic microbes began to recolonize, increasing richness and diversity. Similar observations were reported by Jiang et al. in compost amended with garbage enzyme and biochar (composed of highly active laccase) (Jiang et al., 2024). Notably, on day 5, the MC5 treatment exhibited lower Chao1 and Shannon indices for both bacteria and fungi compared to other treatments, suggesting the emergence of strongly dominant taxa. These likely played important roles in reducing carbon emissions and decomposing organic matter. By the late stage, bacterial richness decreased while evenness increased, whereas fungal richness increased with reduced evenness, indicating that MC5 enriched specific fungal taxa potentially contributing to humification.

**Figure 5:**
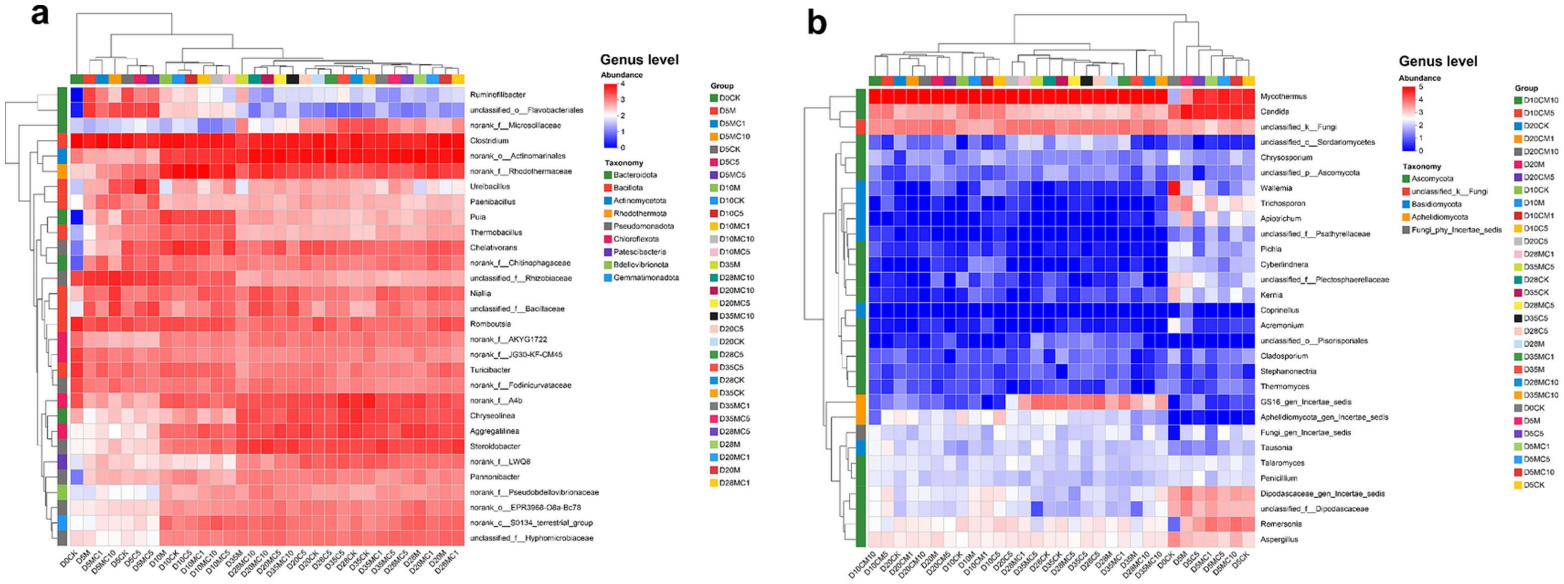
Variations in bacterial **(a)** and fungal **(b)** community abundance under different composting treatments.

Heatmaps visualizing microbial structure at the phylum and genus levels (Figure 5) clearly illustrated the relative abundance and temporal trends of microorganisms under different treatments. The initial mix was dominated by the bacterial phyla *Bacillota*, *Pseudomonadota*, *Bacteroidota*, *Chloroflexota*, and *Actinomycetota*, and the fungal phyla *Ascomycota*, *Basidiomycota*, and *unclassified_k__Fungi*. The relative abundances of these phyla changed significantly over time. In the bacterial domain, *Bacillota*, *Pseudomonadota*, *Bacteroidota*, *Chloroflexota*, and *Actinomycetota* remained dominant throughout, accounting for 79.47–96.47% of all sequences, consistent with previous studies (Peng et al., 2024; Zhang Y. et al., 2025). In the fungal domain, *Basidiomycota* gradually decreased, while *Aphelidiomycota* appeared and increased during the mid to late stages. *Aphelidiomycota*, *Ascomycota*, *Basidiomycota*, and *unclassified_k__Fungi* collectively constituted over 98% of fungal sequences. By day 5, *Bacillota*, *Pseudomonadota*, and *Bacteroidota* had rapidly increased in abundance, becoming the dominant bacterial phyla. These groups are known for their thermotolerance and ability to degrade polysaccharides, cellulose, and lignocellulose during the thermophilic phase (Wang L. et al., 2025). Liang et al. (2024) also noted similar successional patterns in the dominant phyla during co-composting of distillery sludge and waste residue. In the fungal domain, *Ascomycota* became the dominant phylum (more than 80%), playing a key role in degrading recalcitrant organic matter and driving humification, and remained highly abundant until the end of composting.

Initially, the compost feedstock contained a diverse array of microorganisms. No single bacterial genus was dominant, whereas fungi were initially dominated by *Wallemia* and *Candida*. In the first 5 days, abundant soluble organic matter and moderate temperatures supported the survival of many mesophilic microorganisms. By day 5, bacterial composition had not yet changed drastically. After day 10, genera such as *Clostridium*, *norank_o__Actinomarinales*, *norank_f__A4b*, *Aggregatilinea*, *Romboutsia*, and *norank_f__Rhodothermaceae* gradually increased, forming the core bacterial community. *Clostridium* and *Romboutsia* belong to the phylum *Bacillota* and are known to degrade complex organic matter in anaerobic microenvironments within compost (Liu N. et al., 2024; Liu S. et al., 2024; Liu Y. et al., 2024). *norank_f__A4b* and *Aggregatilinea* are members of *Chloroflexota*, capable of participating in organic matter decomposition and nutrient cycling, commonly found in organic-rich environments like compost (Wang et al., 2024). *norank_o__Actinomarinales*, belonging to *Actinomycetota*, can degrade recalcitrant organic compounds; their presence facilitates the breakdown of lignocellulose and promotes compost maturation (Wu et al., 2024a,b). In contrast, fungal communities exhibited clear successional dominance: *Wallemia* was replaced by *Mycothermus* by day 5, and after day 10, a single core fungal genus, *Mycothermus* (76–87%), emerged and persisted until the end of composting. *Mycothermus*, a member of the *Ascomycota*, secretes lignin-degrading enzymes, cellulases, and hemicellulases. It efficiently decomposes polysaccharides in straw, accelerating the composting process and enhancing humification (Xu et al., 2021).

### 3.5 Correlations of dominant genera abundances with environmental factors

**Figure 6:**
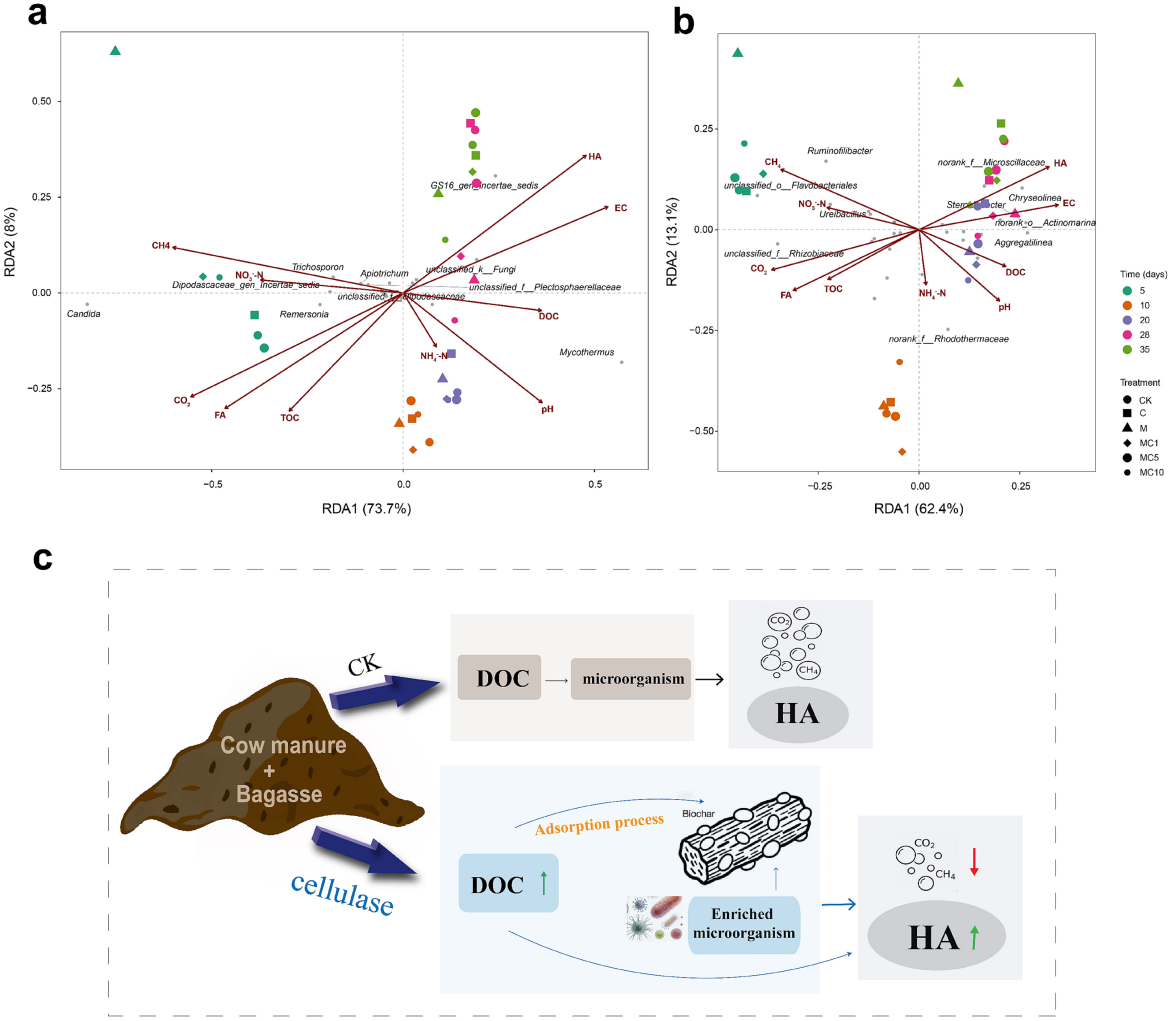
Redundancy analysis (RDA) ordination plots showing the relationships between microbial community structure and environmental factors during composting: **(a)** fungal community; **(b)** bacterial community. Environmental factors include HA, FA, DOC, TOC, EC, pH, NH_4_^+^-N, NO_3_^−^-N, CO_2_ emission, and CH_4_ emission. **(c)** Synergistic effects of biochar and cellulase on carbon emission reduction and humification during co-composting.

To further elucidate the intrinsic mechanisms by which microorganisms regulate carbon transformation, redundancy analysis (RDA) was performed to correlate the bacterial and fungal communities with physicochemical parameters. The results revealed that the fungal community was closely associated with CO₂ emissions and FA (Figure 6a), whereas the bacterial community structure was primarily driven by HA and DOC (Figure 6b). At the fungal level, taxa positively correlated with HA, such as *GS16_gen_Incertae_sedis* and *unclassified_k__Fungi*, were enriched in the MC5 treatment, while those positively correlated with CO₂, including *Candida* and *Remersonia*, were suppressed (Figure 6a). At the bacterial level, key taxa showing significant positive correlations with HA—such as *Chryseolinea*, *norank_f__Microscillaceae*, and *norank_f__A4b*—were enriched in MC5, facilitating the conversion of FA into HA. Conversely, bacterial genera positively associated with CO₂ and CH₄ emissions, including *Clostridium*, *Ruminofilibacter*, and *unclassified_o__Flavobacteriales*, exhibited reduced abundances in MC5, directly providing a microbiological basis for the mitigation of methane and carbon dioxide emissions. Collectively, these findings demonstrate that the MC5 treatment reshapes the microbial community structure, redirecting carbon flow from mineralization pathways toward humification, thereby achieving the dual goals of reducing carbon emissions and enhancing compost quality.

To synthesize the above findings, a conceptual mechanism diagram is presented in Figure 6c. Biochar’s porous structure adsorbs soluble organic carbon, provides microbial habitats, while cellulase hydrolyzes cellulose into fermentable sugars and humic precursors. These actions jointly reshape the microbial community, enriching humification-related taxa and suppressing mineralization-related taxa. Consequently, carbon flow is redirected from greenhouse gas emissions (CO₂/CH₄) toward HA synthesis, achieving carbon emission reduction and compost quality enhancement.

## 4 Conclusion

This study systematically evaluated the effects of biochar and cellulase on the co-composting of cattle manure and bagasse. The combined application of biochar and cellulase demonstrated a significant synergistic effect, outperforming individual amendments in enhancing pile temperature, accelerating the composting process, and increasing the germination index. Furthermore, the combined treatments—particularly MC5—effectively reduced TOC loss and minimized cumulative emissions of CO₂ and CH₄, achieving notable carbon mitigation. The joint application also significantly promoted the conversion of FA to HA, increased HA content, and elevated the HI. These results indicate that biochar and cellulase collectively supply more precursors and create a favorable micro-environment for humic substance synthesis, thereby improving the quality of the final compost product. The microbial mechanism underlying this synergy lies in the restructuring of the microbial community by the combined treatment. Specifically, it enriched key cellulose-degrading genera (such as *Mycothermus*) during the thermophilic phase. Redundancy analysis further revealed that the combined treatment enriched bacterial taxa positively correlated with HA formation (e.g., *Chryseolinea*) and fungal taxa associated with humification (e.g., *GS16_gen_Incertae_sedis*), while suppressing microorganisms linked to greenhouse gas emissions, such as *Clostridium*. These shifts directed organic carbon toward humification rather than mineralization. In summary, the integrated use of biochar and cellulase represents an effective strategy for simultaneously reducing carbon emissions and enhancing humification during composting. This approach holds important implications for advancing the green and sustainable utilization of agricultural waste.

## Data Availability

The datasets presented in this study can be found in online repositories. The names of the repository/repositories and accession number(s) can be found here: NCBI SRA repository, accession number PRJNA1418375.

## References

[ref1] Ahn C. H. Lee S. Park Y. (2024). Particle and bulk media characteristics of food waste compost-based biomedia by dynamic high-temperature aerobic composting process. Environ. Technol. Innov. 35:103711. doi: 10.1016/j.eti.2024.103711

[ref2] Bhatia S. K. Gurav R. Cho D. Kim B. Jung H. J. Kim S. H. . (2023). Algal biochar mediated detoxification of plant biomass hydrolysate: mechanism study and valorization into polyhydroxyalkanoates. Bioresour. Technol. 370:128571. doi: 10.1016/j.biortech.2022.128571, 36603752

[ref3] Bruinenberg M. van Agtmaal M. Hoekstra N. van Eekeren N. (2023). Residues of pesticides in dairy cow rations and fly treatments reduce the number of coleoptera in dung. Agric. Ecosyst. Environ. 344:108307. doi: 10.1016/j.agee.2022.108307

[ref4] Cao Z. Deng F. Wang R. Li J. Liu X. Li D. (2023). Bioaugmentation on humification during co-composting of corn straw and biogas slurry. Bioresour. Technol. 374:128756. doi: 10.1016/j.biortech.2023.128756, 36801442

[ref5] Chang Y. Zhou K. Yang T. Zhao X. Li R. Li J. . (2023). *Bacillus licheniformis* inoculation promoted humification process for kitchen waste composting: organic components transformation and bacterial metabolic mechanism. Environ. Res. 237:117016. doi: 10.1016/j.envres.2023.117016, 37657603

[ref6] Dang R. Cai Y. Li J. Kong Y. Jiang T. Chang J. . (2024). Biochar reduces gaseous emissions during poultry manure composting: evidence from the evolution of associated functional genes. J. Clean. Prod. 452:142060. doi: 10.1016/j.jclepro.2024.142060

[ref7] Du S. Ding S. Wen X. Yu M. Zou X. Wu D. (2024). Investigating inhibiting factors affecting seed germination index in kitchen waste compost products: soluble carbon, nitrogen, and salt insights. Bioresour. Technol. 406:130995. doi: 10.1016/j.biortech.2024.130995, 38885720

[ref8] Geng X. Yang H. Gao W. Yue J. Mu D. Wei Z. (2024). Greenhouse gas emission characteristics during kitchen waste composting with biochar and zeolite addition. Bioresour. Technol. 399:130575. doi: 10.1016/j.biortech.2024.130575, 38479629

[ref9] González D. Barrena R. Moral-Vico J. Irigoyen I. Sánchez A. (2024). Addressing the gaseous and odour emissions gap in decentralised biowaste community composting. Waste Manag. 178, 231–238. doi: 10.1016/j.wasman.2024.02.042, 38412755

[ref10] Gu X. Li H. Shi Y. Li J. Li S. (2024). Regulating bacterial dynamics by mg-modified biochar addition to mitigate gaseous emissions during pig manure composting. J. Clean. Prod. 465:142839. doi: 10.1016/j.jclepro.2024.142839

[ref11] Guo H. Chang Z. Lu Z. Dai Q. Xiang M. Zheng T. . (2024). Enhanced humification of full-scale apple wood and cow manure by promoting lignocellulose degradation via biomass pretreatments. Sci. Total Environ. 929:172646. doi: 10.1016/j.scitotenv.2024.172646, 38653417

[ref12] Huang W. Bao R. Peng Y. Li Q. (2025). Regulation of manganese dioxide on sludge composting to inhibit nitrous oxide generation and promote nitrogen fixation. Chem. Eng. J. 519:165150. doi: 10.1016/j.cej.2025.165150

[ref13] Jiang J. Cui H. Tang Z. Wang S. Cheng K. Zhang C. . (2024). Synergistic effects of biochar and laccase on nitrogen conversation and degradations of two artificial sweeteners during the sewage sludge composting. Chem. Eng. J. 498:155732. doi: 10.1016/j.cej.2024.155732

[ref14] Jiang J. Wang Y. Yu D. Hou R. Ma X. Liu J. . (2022). Combined addition of biochar and garbage enzyme improving the humification and succession of fungal community during sewage sludge composting. Bioresour. Technol. 346:126344. doi: 10.1016/j.biortech.2021.126344, 34780901

[ref15] Jindo K. Sonoki T. Sánchez-Monedero M. A. (2025). Stabilizing organic matter and reducing methane emissions during manure composting with biochar to strengthen the role of compost in soil health. Soil Environ Health 3:100164. doi: 10.1016/j.seh.2025.100164

[ref16] Liang F. Liu X. Yu X. Liu L. He H. Huang C. . (2024). Enhancing bioavailable carbon sources and minimizing ammonia emissions in distillery sludge and distiller's grains waste co-composting through deep eutectic solvent addition. Bioresour. Technol. 397:130491. doi: 10.1016/j.biortech.2024.130491, 38408502

[ref17] Liu Q. He X. Wang K. Li D. (2023). Biochar drives humus formation during composting by regulating the specialized metabolic features of microbiome. Chem. Eng. J. 458:141380. doi: 10.1016/j.cej.2023.141380

[ref18] Liu N. Liu Z. Wang K. Zhao J. Fang J. Liu G. . (2024). Comparison analysis of microbial agent and different compost material on microbial community and nitrogen transformation genes dynamic changes during pig manure compost. Bioresour. Technol. 395:130359. doi: 10.1016/j.biortech.2024.130359, 38272144

[ref19] Liu Y. Wang H. Zhang H. Tao Y. Chen R. Hang S. . (2024). Synergistic effects of chemical additives and mature compost on reducing h2s emission during kitchen waste composting. J. Environ. Sci. 139, 84–92. doi: 10.1016/j.jes.2023.05.030, 38105080

[ref20] Liu S. Zeng J. Cheng Z. He J. Pang Y. Liao X. . (2024). Evaluation of compost quality and the environmental effects of semipermeable membrane composting with poultry manure using sawdust or mushroom residue as the bulking agent. J. Environ. Manag. 353:120162. doi: 10.1016/j.jenvman.2024.120162, 38310794

[ref21] Peng L. Ma R. Jiang S. Luo W. Li Y. Wang G. . (2022). Co-composting of kitchen waste with agriculture and forestry residues and characteristics of compost with different particle size: an industrial scale case study. Waste Manag. 149, 313–322. doi: 10.1016/j.wasman.2022.06.029 35763915

[ref22] Peng W. Wang Y. Cui G. Xu Q. Zhang H. He P. . (2024). Compost quality, earthworm activities and microbial communities in biochar-augmented vermicomposting of dewatered activated sludge: the role of biochar particle size. Biochar 6:6. doi: 10.1007/s42773-024-00365-8

[ref23] Shaibur M. R. Helal A. S. A. Siddique A. B. Husain H. Khan M. W. Sarwar S. . (2025). Cow dung management, biogas production and the uses of bio-slurry for sustainable agriculture. Cleaner Waste Syst. 10:100201. doi: 10.1016/j.clwas.2024.100201

[ref24] Sun C. Wei Y. Kou J. Han Z. Shi Q. Liu L. . (2021). Improve spent mushroom substrate decomposition, bacterial community and mature compost quality by adding cellulase during composting. J. Clean. Prod. 299:126928. doi: 10.1016/j.jclepro.2021.126928

[ref25] Tian X. Qin W. Zhang Y. Liu Y. Lyu Q. Chen G. . (2024). The inoculation of thermophilic heterotrophic nitrifiers improved the efficiency and reduced ammonia emission during sewage sludge composting. Chem. Eng. J. 479:147237. doi: 10.1016/j.cej.2023.147237

[ref26] Wang F. Wang J. He Y. Yan Y. Fu D. Rene E. R. . (2024). Effect of different bulking agents on fed-batch composting and microbial community profile. Environ. Res. 249:118449. doi: 10.1016/j.envres.2024.118449, 38354880

[ref27] Wang L. Wen X. Deng Y. Wei Z. Li J. Song C. (2025). The microhabitat regulation of moisture-ventilation mediated microbial carbon metabolism in response to humic acid formation during chicken manure and food waste composting. J. Environ. Manag. 389:126048. doi: 10.1016/j.jenvman.2025.126048, 40466316

[ref28] Wang Z. Xu Y. Yang T. Liu Y. Zheng T. Zheng C. (2023). Effects of biochar carried microbial agent on compost quality, greenhouse gas emission and bacterial community during sheep manure composting. Biochar 5:5. doi: 10.1007/s42773-022-00202-w

[ref29] Wang J. Zhu N. Zhang J. Shen W. West H. Cao Y. . (2025). Simultaneous reduction of odorous and greenhouse gases emissions by thermophilic microbial agents during chicken manure composting. J. Environ. Manag. 381:125240. doi: 10.1016/j.jenvman.2025.125240, 40199225

[ref30] Wu X. Gao R. Tian X. Hou J. Wang Y. Wang Q. . (2024a). Co-composting of dewatered sludge and wheat straw with newly isolated *xenophilus azovorans*: carbon dynamics, humification, and driving pathways. J. Environ. Manag. 365:121613. doi: 10.1016/j.jenvman.2024.121613, 38944964

[ref31] Wu X. Zhao X. Wu W. Hou J. Zhang W. Tang D. K. H. . (2024b). Biotic and abiotic effects of manganese salt and apple branch biochar co-application on humification in the co-composting of hog manure and sawdust. Chem. Eng. J.:482149077. doi: 10.1016/J.CEJ.2024.149077

[ref32] Xu Z. Qi C. Zhang L. Ma Y. Li G. Nghiem L. D. . (2021). Regulating bacterial dynamics by lime addition to enhance kitchen waste composting. Bioresour. Technol. 341:125749. doi: 10.1016/j.biortech.2021.125749, 34416657

[ref33] Yang Y. Jian Y. He L. (2025). High performance persistent organic pollutants removal using stabilized enzyme aggregates over amino functionalized magnetic biochar. J. Hazard. Mater. 491:137868. doi: 10.1016/j.jhazmat.2025.137868, 40073570

[ref34] Yang X. Yan R. Yang C. Zhang H. Lyu H. Li S. . (2024). Soil accelerates the humification involved in co-composting of wheat straw and cattle manure by promoting humus formation. Chem. Eng. J. 479:147583. doi: 10.1016/j.cej.2023.147583

[ref35] You X. Wang S. Chen J. (2024). Magnetic biochar accelerates microbial succession and enhances assimilatory nitrate reduction during pig manure composting. Environ. Int. 184:108469. doi: 10.1016/j.envint.2024.108469, 38324928

[ref36] Zhang B. Hu X. Guo Z. Qu J. He Y. Han L. . (2025). In-situ remediation efficiency and mechanism of tylosin contaminated soil with biochar immobilized degrading enzyme. J. Hazard. Mater. 497:139483. doi: 10.1016/j.jhazmat.2025.139483, 40914062

[ref37] Zhang Y. Lin B. Hao Y. Lu M. Ding D. Niu S. . (2025). Two-stage inoculation with lignocellulose-degrading microorganisms in composting: enhanced humification efficiency and underlying mechanisms. Environ. Res. 271:120906. doi: 10.1016/j.envres.2025.120906, 39947380

[ref38] Zhang Y. Yang B. Peng S. Zhang Z. Cai S. Yu J. . (2024). Mechanistic insights into chemical conditioning on transformation of dissolved organic matter and plant biostimulants production during sludge aerobic composting. Water Res. 255:121446. doi: 10.1016/j.watres.2024.121446, 38489963

[ref39] Zhao Y. Liu Z. Zhang B. Cai J. Yao X. Zhang M. . (2023). Inter-bacterial mutualism promoted by public goods in a system characterized by deterministic temperature variation. Nat. Commun. 14:5394. doi: 10.1038/s41467-023-41224-7, 37669961 PMC10480208

[ref40] Zhou S. Li Y. Jia P. Wang X. Kong F. Jiang Z. (2022). The co-addition of biochar and manganese ore promotes nitrous oxide reduction but favors methane emission in sewage sludge composting. J. Clean. Prod. 339:130759. doi: 10.1016/j.jclepro.2022.130759

